# Facilitators and barriers to screening for child abuse in the emergency department

**DOI:** 10.1186/1471-2431-12-167

**Published:** 2012-10-23

**Authors:** Eveline CFM Louwers, Ida J Korfage, Marjo J Affourtit, Harry J De Koning, Henriëtte A Moll

**Affiliations:** 1 Department of Public Health, Erasmus MC, University Medical Center Rotterdam, P.O. Box 2040, 3000, CA, Rotterdam, The Netherlands; 2 Department of Pediatrics, Erasmus MC-Sophia Children’s Hospital, Dr. Molewaterplein 60, 3015, GJ, Rotterdam, The Netherlands

**Keywords:** Child abuse, Emergency department, Screening, Qualitative study

## Abstract

**Background:**

To identify facilitators of, and barriers to, screening for child abuse in emergency departments (ED) through interviews with ED staff, members of the hospital Board, and related experts.

**Methods:**

This qualitative study is based on semi-structured interviews with 27 professionals from seven Dutch hospitals (i.e. seven pediatricians, two surgeons, six ED nurses, six ED managers and six hospital Board members). The resulting list of facilitators/barriers was subsequently discussed with five experts in child abuse and one implementation expert. The results are ordered using the Child Abuse Framework of the Dutch Health Care Inspectorate that legally requires screening for child abuse.

**Results:**

Lack of knowledge of child abuse, communication with parents in the case of suspected abuse, and lack of time for development of policy and cases are barriers for ED staff to screen for child abuse. For Board members, lack of means and time, and a high turnover of ED staff are impediments to improving their child abuse policy. Screening can be promoted by training ED staff to better recognize child abuse, improving communication skills, appointing an attendant specifically for child abuse, explicit support of the screening policy by management, and by national implementation of an approved protocol and validated screening instrument.

**Conclusions:**

ED staff are motivated to work according to the Dutch Health Care Inspectorate requirements but experiences many barriers, particularly communication with parents of children suspected of being abused. Introduction of a national child abuse protocol can improve screening on child abuse at EDs.

## Background

Early detection of child abuse is a priority of the Dutch Health Care Inspectorate; in the Netherlands, each year 107,200 children are victim of some type of child abuse [1]. Child abuse is an important public health problem: besides the serious consequences for each child and their environment, the estimated costs of child abuse in the Netherlands are 965 million euros per annum [2,3].

The Dutch media frequently report the inadequate detection of child abuse in hospital emergency departments (EDs). Since January 2009 all EDs are legally required to fulfil the Inspectorate criteria, published in the report ‘*EDs do not adequately detect child abuse: a broken arm is too often an incident*’ in 2008 (Table 1). This report includes a Child Abuse Framework with criteria such as screening each child visiting the ED for child abuse, and regular training for ED staff [4]. Perhaps related to these requirements, the total number of children reported by Dutch hospitals to the central Child Abuse Center increased from 677 (4%) in 2007 to 1,499 (8.3%) in 2010 [5-7].

**Table 1:** Child Abuse Framework of the Dutch Health Care Inspectorate October 2008: all criteria were required to be in place by January 2009

**A. Policy**	
1.	There is policy at the level of the Board of Directors to address child abuse; this policy is documented and funding for this policy is secured.
2.	There is policy within the hospital for dealing with suspected child abuse in the ED. This policy is documented and compliance with policy is checked.
**B. Child abuse team, special child abuse attendant, cooperation with Child Abuse Center**	
3.	A child abuse team is in place. The purpose, duties and procedures of this team are documented. The team has representatives from the ED, a pediatrician, a child psychologist, a social worker and a surgeon; the team meets at least twice a year.
4.	The hospital has a special child abuse attendant who has a job description, and was consulted at least 1–10 times in the first half of 2007. Functionality is ensured by provision of sufficient hours and budget.
5.	Structured consultations take place with the Child Abuse Center; a pediatrician and an ED staff member is present at these consultations. The cooperation is evaluated for procedure and content.
**C. Protocol for suspected child abuse**	
6.	The hospital has a hospital-wide protocol, as well as a protocol in the ED for dealing with signs/suspicions of child abuse. The SPUTOVAMO* checklist and its manual are part of the ED protocol.
**D. Professional development**	
7.	The hospital has a training program for the detection of child abuse. This program is well structured and documented; 95-100% of the ED staff follow the program.
**E. Registry and information**	
8.	It is known how many children visited the ED. The SPUTOVAMO* checklist is used for all (100%) children. These numbers are recorded.
9.	It is known how many children were suspected of child abuse based on the SPUTOVAMO* checklist; these numbers are recorded. A member of staff is available to perform and control these registrations.
10.	For all children who visited the ED in the first half of 2007, it is known how many times the Child Abuse Center was consulted. These numbers are recorded, and for at least 50% of the children of suspected child abuse the Child Abuse Center was consulted.
11.	For all children who visited the ED in the first half of 2007, it is known for how many a referral or report was made to the Child Abuse Center or to other types of aid; these numbers are recorded. Someone is available for implementation and management of this registration.

^*^SPUTOVAMO = Dutch injury registration checklist.

In the present study, ED professionals in Dutch hospitals were interviewed about the quality of child abuse detection in EDs, with the aim to define facilitators/barriers to screening for child abuse, and to make recommendations to optimize the screening for child abuse at EDs.

## Methods

As part of the study *‘Screening for child abuse in EDs, implementation of an optimal protocol’* interviews were held with 27 professionals who were all related to at least one of the seven participating hospitals in the province of South-Holland, the Netherlands [8]. The hospitals included one university (urban) children’s hospital, four urban teaching hospitals, and two rural peripheral hospitals. All participating hospitals had an emergency department where children of all ages were treated. Some of these emergency departments had been undertaken screening for child abuse prior to the staff being surveyed. This period ranged from several years to just one year. At their office, we interviewed members of four professions; nine senior physicians (seven pediatricians and two surgeons), six members of the hospital Board, six ED nurses and six ED managers. These professions were selected because of their direct involvement in the detection of child abuse in the ED or their responsibility concerning child abuse policy. From these 27 interviews, facilitators of and barriers to detection of child abuse were extracted.

In the second phase of the study, these facilitators/barriers were presented to five child abuse experts and one implementation expert for their advice on how to tackle the barriers. These child abuse experts were a pediatrician with expertise in prevention of child abuse, a forensic pediatrician, a child abuse hospital attendant, a forensic nurse specialist in the child abuse detection, and a senior child abuse researcher specialist in child abuse prevention.

All 33 interviews were semi-structured and focused on detection of child abuse in EDs, and related training and policy. All interviewees (except the implementation expert) were also asked for their opinion about ten propositions related to child abuse policy and detection, cooperation, and training. The SPUTOVAMO is a Dutch injury registration checklist developed to detect child abuse in an early stage [9]. All interviews were conducted by the same researcher (EL), all were audio-recorded, and fully transcribed for analysis by two researchers (EL, IK). In 11 interviews a second researcher was (IK or MA) present. Reasons for this were twofold: to train the first interviewer (EL) and to underline the importance of some of the interviews: these were the interviews with six members of the hospital Board, and with the implementation expert.

This study was approved by the Medical Ethical Committee of the Erasmus MC, University Medical Centre Rotterdam (MEC-2007-195). Participants were professionals and informed consent for participation was audio-recorded.

## Results

The 33 interviews (conducted between June 2007 and January 2008) lasted on average 38 (range 22–76) minutes each.

First, the health professionals were asked if they ever suspected child abuse in the ED and what they found difficult about these situations. Four of the seven pediatricians found it difficult to discuss suspected child abuse with the parents; this was mainly due to practical problems (e.g. limited time, lack of a suitable/quiet location) and personal barriers (e.g. fear of an unjustified suspicion). The two surgeons had a similar experience and also mentioned the problem of separating the child’s medical treatment from the investigation of possible abuse. They considered medical treatment to be their prime responsibility and prefer to leave investigation of abuse to other professionals, e.g. the pediatrician or the Child Abuse Center (Table 2, proposition 2). Five ED nurses considered communication to be a limiting factor, e.g. when parents questioned the need for a head-to-toe examination when their child had a local injury only.

**Table 2:** Propositions presented to the interviewees at the end of the interview

**Propositions A. Policy**	**Agree**	**Disagree**	**No opinion**
1. It is better to have an unjustified suspicion than to miss a case of child abuse (n=32)	30	2	0
2. Other specialties are pleased to let the pediatrician conduct the discussion with parents in the case of suspected child abuse (n=32)	25	1	6
3. Sometimes I do not report a suspicion of child abuse in order to avoid problems with the parents (n=26; not presented to members of the Board)	10	15	1
**Propositions B. Child abuse team, special child abuse attendant, cooperation with Child Abuse Center**	**Agree**	**Disagree**	**No opinion**
4. The Child Abuse Center is sufficiently accessible for reporting child abuse (n=26; not presented to members of the Board)	15	3	8
5. When it comes to child abuse, patient privacy is subordinate to the interests of consultations between health professionals (n=32)	23	6	3
**Propositions C. Protocol for suspected child abuse**	**Agree**	**Disagree**	**No opinion**
6. In our ED more than 90% of the child abuse cases are detected (n=32)	3	23	6
7. If no follow-up is organized, you might as well stop screening for child abuse (n=32)	16	16	0
8. Our ED staff is well informed about when/when not to fill out a screening instrument for child abuse (n=32)	16	9	7
**Propositions D. Professional development**	**Agree**	**Disagree**	**No opinion**
9. My medical training was sufficient to enable me to detect child abuse in practice (n=26; not presented to members of the Board)	3	20	3
10. Prejudice precludes proper detection of child abuse (n=32)	24	8	0

These answers are derived from 32 interviewees (i.e. excluding the implementation expert), or from 26 interviewees (i.e. excluding the implementation expert and the 6 Board members).

### Child abuse framework

During the interviews, the following elements of the Inspectorates’ Child Abuse Framework (Table 2) were mentioned.

A. **Policy** (propositions 1–3): Health professionals saw active support from the hospital Board as a positive factor, whereas the lack thereof was seen as a bottleneck. When the Board was supportive they arranged for example the appointment of a special child abuse attendant. The Board unanimously indicated that they were open to a more active policy on the detection of child abuse. However, one Board member remarked: ‘*It’s difficult to find budgeting in these times of cutbacks*’ and another said: ‘*We can tackle all sorts of problems of our society but if there are no financial compensations, then we should really limit to our core business; treating real pathology.’*

B. **Child abuse team, child abuse attendant, collaboration Child Abuse Center** (propositions 4, 5): Three of the 7 hospitals had a child abuse team which focused on policy and/or cases. Organizing a team meeting was a bottleneck ‘…*because it’s difficult to meet during working hours and people aren’t so willing to meet after work’*. Five Board members found the appointment of a child abuse attendant useful, but ‘…*no money was available*’, or ‘*it belongs to the normal package of social work’*. One Board member was ‘…*not in favor of creating functions with special areas, as the primary person (ED nurse) would no longer feel responsible’*.

The health professionals were satisfied with the collaboration with the Child Abuse Center.

C. **Protocol for suspected child abuse** (propositions 6–8): All physicians stated that their hospital had a protocol for suspected child abuse. However, among the other interviewees, not all were aware of it or did not know where to find the protocol.

At the time of the interviews, screening for child abuse by completing a SPUTOVAMO form (or a checklist derived from SPUTOVAMO) was conducted in 5 of 7 participating hospitals; 2 hospitals did not screen for child abuse because of disagreement about its usefulness or about the profession that should complete the screening instrument. Irrespective of whether or not screening took place, the majority thought that child abuse is not always detected in the ED. ED managers agreed that screening belongs to the work of the ED. However, during busy hours ED nurses often disregard the checklist, even though it can be filled in relatively quickly.

D. **Professional development** (propositions 9, 10): In all hospitals the pediatricians provided some training on recognizing and dealing with child abuse, albeit sporadically and without a structured program. In one hospital, all staff had recently received intensive training in detecting child abuse. A fast turnover of ED staff (especially junior doctors) was an obstacle to organizing teaching and maintaining the level of knowledge. Two physicians found that lack of motivation among the ED staff was also an obstacle. Almost all nurses and physicians stated that more emphasis should be placed on detecting child abuse during their basic training.

### Expert opinions

Also interviewed were five child abuse experts and in addition, we asked an implementation expert for advice on how to implement a screening protocol for child abuse at EDs.

A. **Policy:** To ensure funding for the policy to tackle child abuse, two experts advised to adjust the DBC code (Diagnostic/Treatment code in the Dutch medico-financial system) for child abuse ‘…*then hospitals will receive the money they need for this type of care’.*

B. **Child abuse team, child abuse attendant, collaboration with the Child Abuse Center:** The experts think that child abuse teams are necessary for good collaboration between the various disciplines. Two experts advised to evaluate the policy twice a year with the complete team; for specific cases they advised to review these only with the specific professionals involved. Four experts found it worthwhile to invest in and appoint an attendant specifically for child abuse, especially because psychosocial research and referral to child care entails considerable time and effort. A child abuse attendant can guarantee quality control, rapidity of treatment or referrals, and proper follow-up of patients.

C. **Protocol for suspected child abuse:** Introduction of a national protocol, with local modifications, was supported by the experts. This will ensure uniformity of the process and prevent each hospital having to develop its own protocol.

All experts found screening for child abuse at EDs worthwhile, and considered a head-to-toe examination an essential part of screening, because important signs of child abuse often can be found on the skin. This is not standard practice for all ED nurses, because they often have a problem with undressing a child completely when the child has only a local complaint or injury. Overall screening for child abuse can become more acceptable for ED nurses and parents if the hospital informs all parents about the routine screening process, e.g. via brochures, flyers, announcements, etc.

D. **Professional development:** The experts emphasised that for successful screening and early detection of child abuse, ED staff needs adequate training. This can be realized by including detection of child abuse in the medical training of physicians and nurses; in this way physicians will also learn to include child abuse in their differential diagnosis. Important topics during training are interviewing techniques/communication skills, and relating injuries with the history and developmental phase of the child.

#### Implementation expert

When implementing improvements in a workplace, it is important to proceed along appropriate steps. The following steps are based on the model of Grol et al. [10,11].

The first step is to define ‘good care’ based on the literature and/or expert opinions. Then, indicators are defined to measure the quality of good care, e.g. ‘…*during the triage ED nurses will screen for child abuse in more than 90% of the children’*. Subsequently, the current situation is investigated in the participating hospitals, i.e. do they meet the indicators of good care? If not, the barriers to this are explored by means of interviews or questionnaires. A decision is made as to which part of the implementation package is needed in each hospital, and implementation can then start. Finally, the effect can be measured by the indicators of good care.

The facilitators and barriers for screening of child abuse at emergency departments are summarized in Table 3.

**Table 3:** Facilitators and barriers for screening of child abuse in emergency departments

**Facilitators**	**Barriers**
Support of the Hospital Board	Practical problems (e.g. limited time)
Presence of child abuse attendant	Personal barriers (e.g. fear of an unjustified suspicion)
Presence of child abuse team	Insufficient communication skills
Intensive training of ED staff	Fast turnover of ED staff
Financial support	

## Discussion

Since January 2009 Dutch hospitals are legally required to fulfill the criteria of the Child Abuse Framework of the Dutch Health Care Inspectorate [4]. Most of the hospitals in the present study met most of these criteria. In general, this was promoted by a supportive Board, the presence of a child abuse attendant, a protocol for suspected child abuse or an appropriate screening instrument. However, many barriers to adequate detection of child abuse at EDs still exist. More time, money and effort of health professionals and management are needed to tackle these barriers. Previous studies have shown that screening for child abuse in emergency departments is effective to increase the detection of suspected child abuse, but a validated protocol or screening instrument is lacking [8,12-15].

Health professionals are motivated to improve the detection of child abuse, but lack sufficient time to develop adequate policy and protocols, to register (suspicions of) child abuse, and to organize education and training. Moreover the ED’s high patient flow with its great diversity in severity of symptoms, makes it hard for ED staff to calmly discuss a suspicion of child abuse with parents. The appointment of a dedicated child abuse attendant who can perform all these tasks could be a solution. Unfortunately, not all hospital directors, whose support is needed to create such a function, are convinced of this necessity. The Inspectorate sees the appointment of a child abuse attendant as a condition of delivering responsible care [4]. In addition to a child abuse attendant, a child abuse team will promote the signaling and detection of (suspected) child abuse [16].

Implementation of a national screening protocol, including a screening instrument applicable for all children and an appropriate procedure for situations when child abuse is suspected, is required but not yet available [14]. Developments are ongoing and the validity of various screening instruments is currently being investigated.

None of the participating hospitals had a structured training program for the detection of child abuse or for the care of abused children. The design of such a program is impeded by the high turnover of (especially) junior doctors in EDs. Nevertheless, it is essential to develop such programs, because education is the basis for proper detection of child abuse. In addition, effective interviewing techniques can lower the threshold to discuss suspicions of abuse with parents [4,14]. Management support is essential to realize structured training programs. In the Netherlands there are good opportunities for this, e.g. e-learning for ED nurses, and a two-day course for physicians are available [17,18].

Detailed registration of the numbers and types of suspected child abuse cases in hospitals is important. This can be largely automated and integrated with the electronic patient file. Then, based on these data, the extent of the workload (part-time/full-time) for a child abuse attendant can be calculated, as well as other requirements, e.g. the need for consulting hours for suspected cases of child abuse.

A limitation to be mentioned for this study is that the interviews were conducted before the Health Care.

Inspectorate published its report, and some topics that were addressed in the report, such as registration and information were not addressed in our interviews [4]. Because we wanted to compare perspectives from different disciplines we interviewed professionals of mixed background. A limitation of this approach is that we interviewed small numbers per discipline.

At the beginning of our study screening for child abuse had been ongoing in some of the participating emergency departments while others had not even started, which is also a limitation of this study.

## Conclusions

In summary, the health professionals in the present study are motivated to adhere to the Child Abuse Framework of the Health Care Inspectorate, but experience many barriers. When child abuse is suspected, communication is often the main bottleneck. Management should create opportunities, such as adequate training and appointment of a child abuse attendant, to enable health professionals to better commit themselves to improved detection of child abuse. Simply acknowledging the problems and approving the policy is not sufficient. Implementation of a national protocol for suspected child abuse, including relevant training and a validated screening instrument, will go a long way to removing these barriers.

## Abbreviations

ED: Emergency departments; SPUTOVAMO: A Dutch injury registration checklist; DBC code: Diagnostic/Treatment code in the Dutch medico-financial system; Child Abuse Centre: the competent authority in the Netherlands that is responsible for taking care of cases of (potential) child abuse. They explore the cases of suspected child abuse and take care of adequate aid if necessary. All kinds of professionals as well as citizens can voluntary report suspected child abuse to the CAC, there is no mandatory reporting in the Netherlands; Child Abuse Teams: multidisciplinary teams in hospitals that deal with child abuse policy and assist hospital staff when child abuse is suspected.; Forensic paediatrician and nurse: experts in the field of forensic pediatrics, working at a national independent forensic institute.

## Legend of terms

ED: Emergency departments; SPUTOVAMO: A Dutch injury registration checklist; DBC code: Diagnostic/Treatment code in the Dutch medico-financial system; Child Abuse Centre: the competent authority in the Netherlands that is responsible for taking care of cases of (potential) child abuse. They explore the cases of suspected child abuse and take care of adequate aid if necessary. All kinds of professionals as well as citizens can voluntary report suspected child abuse to the CAC, there is no mandatory reporting in the Netherlands; Child Abuse Teams: multidisciplinary teams in hospitals that deal with child abuse policy and assist hospital staff when child abuse is suspected.; Forensic paediatrician and nurse: experts in the field of forensic pediatrics, working at a national independent forensic institute.

## Competing interests

This study was funded by the Netherlands Institute for Health Research and Development (ZonMw 63 30 00 23). The authors were completely independent from funders in conducting this study and writing this manuscript.

## Authors’ contributions

EL designed and carried out the interviews, analyzed the data and drafted the manuscript. IK designed and participated in the interviews, analyzed the data and helped to draft the manuscript. MA participated in the interviews. HK and HM interpreted the analysis. All authors read and approved the final manuscript.

### Legend of terms

Child Abuse Centre, the competent authority in the Netherlands that is responsible for taking care of cases of (potential) child abuse. They explore the cases of suspected child abuse and take care of adequate aid if necessary. All kinds of professionals as well as citizens can voluntary report suspected child abuse to the CAC, there is no mandatory reporting in the Netherlands; Child Abuse Teams, multidisciplinary teams in hospitals that deal with child abuse policy and assist hospital staff when child abuse is suspected.; Forensic paediatrician and nurse, experts in the field of forensic pediatrics, working at a national independent forensic institute.

### Authorship

All authors confirm that they have read and approved the paper, and that they have met the criteria for authorship as established by the International Committee of Medical Journal Editors.

## Pre-publication history

The pre-publication history for this paper can be accessed here:

http://www.biomedcentral.com/1471-2431/12/167/prepub
